# Mapping the literature employed to teach law in universities in Anglophonic and Hispanophone countries in the nineteenth century to explore the circulation of legal ideas: Protocol for a scoping review

**DOI:** 10.1371/journal.pone.0353421

**Published:** 2026-10-27

**Authors:** Paula Aguilar Peña, Fernando Muñoz León

**Affiliations:** 1 Facultad de Educación, Universidad San Sebastián, Concepción, Chile; 2 Facultad de Derecho y Ciencias Sociales, Instituto de Historia, Universidad San Sebastián, Concepción, Chile; PLOS: Public Library of Science, UNITED KINGDOM OF GREAT BRITAIN AND NORTHERN IRELAND

## Abstract

The literature used for teaching law in universities during the nineteenth century played a decisive role in shaping legal cultures and facilitating the transnational circulation of legal ideas. However, existing research on this topic remains fragmented across disciplines, time, regions, and historiographical traditions. This scoping review aims to systematically map and synthesize the existing academic literature on the books used for teaching law in universities in Anglophonic and Hispanophone countries during the nineteenth century. Following the Joanna Briggs Institute (JBI, Adapted from JBI. *JBI scoping review template*. Adelaide: Joanna Briggs Institute; 2020. Available from: https://jbi.global/ebp) guidelines, the review will identify and categorize studies addressing what law manuals, treatises, and other pedagogical texts were employed, as well as the scholarly interpretations of their circulation and use. By gathering the literature that addresses the research question, “What books were employed to teach law in universities in Anglophonic and Hispanophone countries in the nineteenth century?”, we hope to assess the scope of existing research and provide a structured foundation for future studies in intellectual history and the history of universities. This article presents the a priori protocol detailing the planned methodology for conducting the Scoping Review.

## Introduction

The study of the literature used for teaching and studying law in universities during the nineteenth century constitutes a privileged avenue for understanding the formation of modern legal cultures and the processes of transnational circulation of legal ideas. Manuals, treatises, compendia, and other pedagogical texts not only organized access to legal knowledge for generations of students, but also functioned as central vehicles for the translation, adaptation, and reinterpretation of doctrines produced in different national and imperial contexts. In this sense, the analysis of books and texts used in university legal education allows us to observe, in an empirically grounded manner, how legal concepts, categories, and traditions moved across time and space.

During recent decades, various historiographical approaches have examined the role of textbooks and manuals in shaping professional legal culture, both in Europe and the Americas, and have emphasized the importance of attending to the material and pedagogical supports of legal knowledge. Related studies have explored curricula [1–6], teaching, discursive, and writing practices [7–27], and the transnational transfer of doctrines [28–33], presenting a wide range of conclusions across different national and institutional contexts [34–45]. These approaches have shown that teaching texts cannot be understood as mere passive reflections of abstract doctrines, but rather as cultural artifacts produced and used within specific institutional contexts, shaped by editorial, curricular, and regulatory decisions.

However, research on the literature used to teach law in the nineteenth century in these regions remains fragmented and dispersed across multiple disciplinary traditions and linguistic areas, which hinders the construction of an overall view of the field and, consequently, a comprehensive understanding of how pedagogical legal texts functioned as vectors of intellectual exchange during the nineteenth century. To date, in particular, there is no consolidated mapping of research specifically devoted to the books used for teaching law in universities in Anglophonic and Hispanophone countries during this period.

In line with the methodological guidelines of the JBI, preliminary searches were conducted in Web of Science, Scopus, and Google Scholar in order to identify systematic reviews, scoping reviews, or other research syntheses related to nineteenth-century legal teaching literature. These searches yielded only a limited number of reviews partially connected to the topic, generally focused on specific national contexts or different time periods. Indeed, only a few literature reviews have been identified that cover loosely defined temporalities and specific regions [46,47]. Taken together, these limitations reveal the absence of a comprehensive, comparative, and methodologically robust mapping of the field, thus justifying the need for the present scoping review.

Accordingly, this scoping review aims to map and synthesize the available research on the books used for teaching law in universities in Anglophonic and Hispanophone countries during the nineteenth century. Preliminary searches indicate that there exists a sufficiently diverse corpus of studies from legal history, the history of universities and higher education, as well as related fields, making it possible to develop a review aligned with the Population–Concept–Context (PCC) framework guiding this protocol.

The general objective of this scoping review is to identify, describe, and categorize existing studies on the literature used in university legal education in the nineteenth century in English- and Spanish-speaking countries, in order to identify approaches, trends, and opportunities for future research. The review will include empirical studies with qualitative, quantitative, or mixed-method designs, as well as theoretical works relevant to the conceptualization of the phenomenon. Opinion pieces, editorials, and non-academic commentary will be excluded.

### Research question

What books were used for teaching law in universities in Anglophonic and Hispanophone countries in the 19th century?

### Inclusion criteria

#### Population.

This scoping review will include studies addressing universities and other higher education institutions in Anglophonic and Hispanophone countries during the nineteenth century. Research focusing on universities, law schools, or other equivalent academic units responsible for higher legal education will be considered. Eligible studies may focus on one or multiple institutions, countries, or regions, as long as they explicitly address the teaching of law in the university context of the period under study.

#### Concept.

The central concept of this review is the literature used for teaching law in universities in Anglophonic and Hispanophone countries during the nineteenth century, understood as the set of manuals, treatises, compendia, lecture texts, and other books used for pedagogical purposes in higher legal education. Studies that describe, analyze, or theorize about the production, circulation, adaptation, or uses of these texts in university legal training will be included. Research focused exclusively on doctrinal or intellectual production about law without reference to its use in teaching contexts will be excluded.

#### Context.

The review will consider studies conducted in the context of universities in Anglophonic and Hispanophone countries during the nineteenth century. Only publications in English and Spanish will be included, in accordance with the linguistic competencies and areas of expertise of the research team.

#### Types of sources.

This scoping review will consider empirical studies reporting findings on the use of legal literature in university teaching. Following JBI guidelines, a wide range of research designs will be included to comprehensively map the available evidence. Quantitative, qualitative, and mixed-methods studies will be considered. Reviews, editorials, and opinion articles will be excluded; however, theoretical works that conceptualize, describe, or propose analytical frameworks relevant to the study of legal teaching literature will be included. Reference lists of relevant studies and reviews will be examined to identify additional eligible literature.

## Methods

A scoping review will be conducted following the methodological guidance of the JBI manual, with the aim of identifying and mapping the types of published evidence available in the Humanities and Social Sciences regarding legal manuals used in higher education in the nineteenth century. Their characterization will allow us to explore how the circulation of legal ideas developed during that period. In this way, the study will summarize and organize the available evidence, making this body of knowledge accessible. Indeed, the purpose of this scoping review is to provide an overview of the available evidence [48] in order to contribute to knowledge and understanding of the development of legal education.

### Search strategy

The search strategy will consist of identifying studies whose results have been published in academic articles, using a three-stage approach:

1. An initial limited exploratory search was conducted in Google Scholar to identify relevant published articles and to investigate previous reviews in the field. The following keywords were used (Table 1):

**Table 1:** Keywords.

	*Population*	*Concept*	*Context*
*Spanish*	*Profesores y estudiantes de Derecho en el contexto de la educación superior.*	*Literatura empleada para la enseñanza del derecho en las universidades de países angloparlantes e hispanohablantes en el siglo XIX para explorar la circulación de ideas jurídicas.*	*Universidades y facultades de derecho en el siglo XIX en países angloparlantes e hispanohablantes.*
*English*	*Law professors and students in the context of higher education*	*Literature employed for teaching law in universities in Anglophonic and Hispanophone countries in the 19th century to explore the circulation of legal ideas*	*Universities, Law Schools, in the 19th Century in Anglophonic and Hispanophone countries*

Source: Prepared by the authors

2. Second, the strategy will be adapted by designing a specific Boolean search string for each database (Web of Science, Scopus, and Ebscohost). Example (Scopus);

(“law manual*” OR “legal textbook*” OR “legal treatise*” OR “handbook* of law”) AND (“academic discussion” OR “theoretical framework” OR “legal pedagogy” OR “legal education”) AND (“America*” OR “Europe*” OR “United States” OR “Latin America” OR “Germany” OR “France” OR “Spain”) AND (“19th century” OR “nineteenth century” OR “1800s”)

3. Third, the reference lists of included sources will be reviewed to identify additional studies that may not have been included in the data base previously constructed.

**Sources of information:** Only studies published in English and Spanish will be included due to the linguistic competencies of the researchers. No limits on publication date will be applied, as the aim is to achieve a broad temporal identification of studies in this field.

### Study selection

All citations will be collected and uploaded into Rayyan reference management software, and duplicates will be removed. After a pilot test, titles and abstracts will be independently screened by the two members of the research team. Disagreements or uncertainties will be resolved through discussion, and if necessary, a third external reviewer will be consulted. The inclusion process will be presented in the final review using a PRISMA flow diagram.

Potentially relevant sources will then be assessed through full-text analysis using Dedoose qualitative analysis software, and relevant data will be extracted from each source.

### Data extraction

Data will be extracted by two reviewers using a tool developed by the authors, summarized in the following table (Table 2):

**Table 2:** Data extraction.

*APA 7th edition reference*	*Objective*	*Participants and sample size*	*Methodological design*	*Proposals*	*Suggestions or discussion*

Source: Prepared by the authors

If modifications to categories emerge, they will be reported. If some data are missing, additional information will be sought from the authors.

### Data analysis and presentation

The results will be presented narratively, supported by multimodal resources such as visual elements, charts, and diagrams. This visual presentation is relevant because meaning in a text emerges from the systematic interaction between verbal, graphical, mathematical, and typographic systems [49,50]. The narrative summary will explain how the results relate to the research objectives and question.

### Study status

At the time this protocol was submitted, the scoping review remained at a preliminary stage and had not yet yielded results. Initial exploratory searches were undertaken in order to refine the search strategy and evaluate the viability of the proposed review topic. Nevertheless, the stages involving formal database searches, study selection, data extraction, and analytical procedures had not yet commenced. Comprehensive searching and screening activities are scheduled to start in July 2026 and are expected to conclude by October 2026. The processes of data extraction and synthesis are projected to continue through December 2026. Submission of the results for publication is anticipated during 2027.

## Discussion

A systematic mapping of existing studies is therefore indispensable to clarify what is currently known about the literature used for teaching law in universities during the nineteenth century, as well as the theoretical approaches employed in its analysis. This step is necessary to understand both the patterns of circulation of legal ideas in existing research. A scoping review emerges as the most appropriate method for this purpose, as it allows for the systematic exploration of a broad and heterogeneous body of literature. This material is fundamental because, as we have stated, the literature used for teaching law in universities during the nineteenth century played a decisive role in shaping legal cultures and facilitating the transnational circulation of legal ideas.

## Supporting information

S1. File PRISMA-P (Preferred reporting items for systematic review and meta-analysis protocols) checklist. (PDF)
